# GNSS-Based Narrow-Angle UV Camera Targeting: Case Study of a Low-Cost MAD Robot

**DOI:** 10.3390/s24113494

**Published:** 2024-05-28

**Authors:** Ntmitrii Gyrichidi, Alexey M. Romanov, Oleg V. Trofimov, Stanislav A. Eroshenko, Pavel V. Matrenin, Alexandra I. Khalyasmaa

**Affiliations:** 1 Institute of Artificial Intelligence, MIREA—Russian Technological University (RTU MIREA), 119454 Moscow, Russia; girikhidi0@gmail.com (N.G.);; 2 Ural Power Engineering Institute, Ural Federal University Named after the First President of Russia B.N. Yeltsin, 620002 Ekaterinburg, Russia; s.a.eroshenko@urfu.ru (S.A.E.); matrenin.2012@corp.nstu.ru (P.V.M.); a.i.khaliasmaa@urfu.ru (A.I.K.); 3 Power Supply Systems Department, Novosibirsk State Technical University, 630073 Novosibirsk, Russia

**Keywords:** UV sensors, corona discharge, global navigation satellite system, real-time kinematic, energy, power transmission line, substation, inspection, camera targeting

## Abstract

One of the key challenges in Multi-Spectral Automatic Diagnostic (MAD) robot design is the precise targeting of narrow-angle cameras on a specific part of the equipment. The paper shows that a low-cost MAD robot, whose navigation system is based on open-source ArduRover firmware and a pair of low-cost Ublox F9P GNSS receivers, can inspect the 8 × 4 degree ultraviolet camera bounding the targeting error within 0.5 degrees. To achieve this result, we propose a new targeting procedure that can be implemented without any modifications in ArduRover firmware and outperforms more expensive solutions based on LiDAR SLAM and UWB. This paper will be interesting to the developers of robotic systems for power equipment inspection because it proposes a simple and effective solution for MAD robots’ camera targeting and provides the first quantitative analysis of the GNSS reception conditions during power equipment inspection. This analysis is based on the experimental results collected during the inspection of the overhead power transmission lines and equipment inspections on the open switchgear of different power plants. Moreover, it includes not only satellite, dilution of precision, and positioning/heading estimation accuracy but also the direct measurements of angular errors that could be achieved on operating power plants using GNSS-only camera targeting.

## 1. Introduction

For the last decade, multiple robots for the non-destructive inspection of high-voltage power equipment have been introduced [1,2,3,4,5]. Most of these robots combine a mobile platform and a pan-and-tilt rotation device to target one or several cameras. These cameras automatically read out parameters from different indicators and perform multi-spectral diagnostics of power equipment [1]. Generally, the inspection mission can be presented as a set of keypoints, in which the robot sequentially targets its cameras on specific parts of equipment and performs data acquisition [6].

To maximize the resolution of the recorded images and video sequences, engineers often use cameras with narrow angles (high zoom ratio). When our Multi-Spectral Automatic Diagnostic (MAD) robot design was presented at the BUSSEC IEEE conference [6,7], it sparked an intensive discussion about whether the narrow-angle ultraviolet (UV) camera is critical for power equipment inspection and if there is a possibility to use wide-angle cameras to maximize the field of view (FOV) and significantly simplify the camera targeting. Thus, we decided to illustrate the importance of a narrow-angle UV camera in the inspection application with an example.

**Figure 1:**
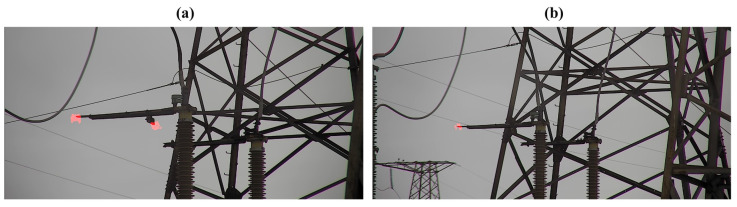
The example of the same corona discharge activity recorded from the distances of (**a**) 28 m and (**b**) 37 m using the Ofil Rail HD UV camera with the same UV channel gain. The images are generated by combining a frame from the RGB channel with the average corona activity registered by the UV channel during 9 s periods (red zones).

Due to the high cost of UV cameras for corona discharge activity registration, we were unable to undertake two recordings from the same point of the same power equipment using two cameras with identical sensors but different FOVs. Instead, we recorded the same high-voltage disconnect switch (the contacts of the opened disconnect switch are natural sources of corona discharge activity) from the distances of 28 m and 37 m, which is similar to increasing the FOV by approximately 32% in terms of the UV intensity registered by each pixel of the camera sensor. In both cases, we used a RailHD camera with an 8×4° FOV, which recorded data for 9 s using the same UV channel gain. As observed in Figure 1, even such a slight change in the zooming factor results in the loss of the corona discharge source located on the right part of the disconnect switch. The only way to improve the situation is to increase the UV channel gain. However, that will cause more noise, thus making it difficult to distinguish the corona discharges caused by the potential defects.

Moreover, the increase in FOV reduces the inspected part’s resolution in both the UV and RGB channels, making it challenging to locate the actual source of the corona activity correctly. Thus, UV cameras with FOVs of, e.g., 20–25°, will result in significantly higher risks of missing defects in their initial state. A partial solution for cameras with relatively high FOVs (compared to Ofil RailHD) is to record the data on much shorter distances. However, in this case, due to the standard general arrangement of the substation sites, other equipment often overlaps the inspection point of interest, located on the camera’s line of sight.

**Figure 2:**
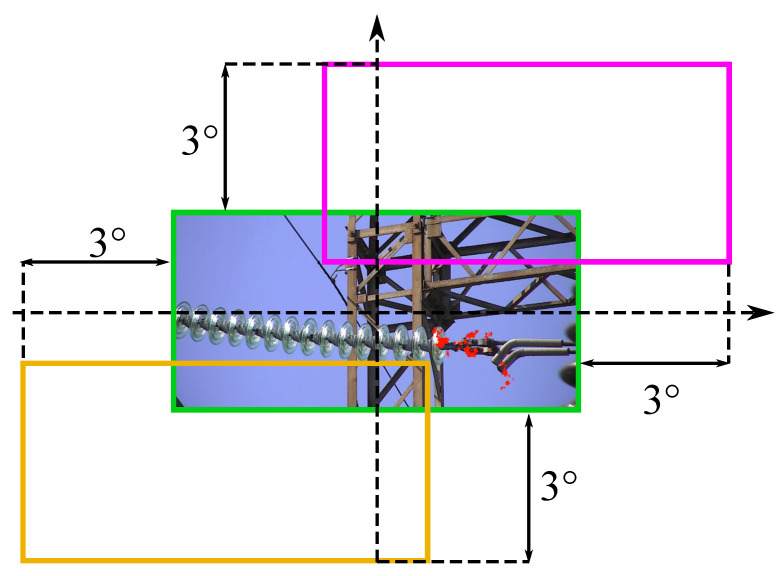
The boundaries of 8×4° UV camera frame in a case of targeting error. The green line is a boundary of the original frame (red dots—immediate UV corona discharge activity). Orange line—the frame boundary in case of −3/−3° pan/tilt error. Magenta line—the boundary of the frame in case of 3/3° pan/tilt error.

Despite all the advantages of narrow-angle UV camera usage from the inspection point of view, it significantly increases the requirements for targeting precision and repeatability. Even the slightest deviations in position and heading estimates lead to the disappearance of the parts of the equipment under test from the camera’s FOV. The last statement is illustrated with Figure 2, which shows an inspection image captured by an 8×4° UV camera [6].The green line shows the area of the original frame. The inspection robot’s targeting error may reach up to 3° [8]. The orange and the magenta frames represent the camera’s FOV in the cases of −3/−3° and 3/3° pan/tilt error. As observed in both cases, the camera will miss the defect (the first insulator disk with corona discharge activity). At the same time, as mentioned before, increasing the FOV will reduce the UV sensor’s resolution and sensitivity.Thus, the precise targeting of the narrow-angle camera is one of the key challenges in MAD robot design.

Modern inspection robots are equipped with complex navigation systems that fuse multiple sensors: Global Navigation Satellite System (GNSS) receivers, Light Detection and Ranging sensors (LiDARs), cameras, odometry, etc. [1,4], to fulfill these requirements. At the same time, according to [9], modern low-cost GNSS receivers working in real-time kinematic (RTK) positioning mode can already provide enough precision for robot positioning and camera targeting. Also, multiple recent papers in the field of agricultural robotics report successful cases of precise navigation using the inertial measurement unit (IMU) and GNSS RTK as the only sources of navigation data [10,11,12]. Thus, it is potentially possible to reduce the cost of power equipment inspection robots by removing LiDARs, navigation cameras, and the corresponding embedded computers used to process these sensors, solving all the navigation and targeting tasks using a GNSS RTK and IMU. At the same time, it is not fair to directly disseminate the result achieved by agricultural robots to other application areas. Contrary to the field or the garden, in a power plant or a substation, many metal constructions around the robot, including a net of power lines above the GNSS antennas, may influence the reception of satellite radio signals. Moreover, the electromagnetic noise from the high-voltage equipment significantly reduces the quality of the magnetometer’s output [13], which is generally not an issue in agricultural applications. The influence of these factors on the GNSS-based navigation and camera targeting of the power equipment inspection robot requires additional investigation.

This research aims to fill the gap mentioned above by performing an analysis of the inspection results’ repeatability achieved by the MAD robot equipped with a narrow-angle (8×4°) UV camera and a low-cost GNSS RTK navigation system. The studies were performed on two different power plants and in the field during the inspection of overhead power lines. The main contributions of the research to the state of the art are the following: (1) quantitative results representing the GNSS RTK operational conditions during the inspection of the power equipment, which demonstrate that the conditions of the satellite signal reception on a substation are similar or even better than in vineyards (to the best of our knowledge, it is the first quantitative analysis of the GNSS RTK quality on high-voltage facilities); (2) a new GNSS-based camera targeting method that can be implemented using standard ArduRover firmware, with increasing repeatability up to 2.5 times and bounding targeting error within ±0.5°; (3) direct measurements of angular errors that could be achieved on operating power plants using GNSS-only camera targeting; (4) comparison results showing that a GNSS RTK combined with IMU is sufficient for the narrow-angle camera targeting during power equipment inspection and outperforms other navigation approaches, including LiDAR simultaneous localization and mapping (SLAM) and ultra-wideband beacons (UWB), in this task.

All the results of this research have been achieved using Ublox F9P multi-band GNSS RTK receivers and the CUAV X7 navigation controller running the open-source ArduRover firmware. Using such low-cost, commercially available components makes these results relevant to various research and engineering projects in robotics and energy system inspection.

The rest of the paper is organized as follows. Section 2 provides a brief review of the related works. Section 3 describes the robot and the experimental sites used in the current research. The GNSS reception quality and positioning accuracy at different experimental sites are analyzed in Section 4. The GNSS camera targeting methods and their repeatability are introduced in Section 5. Section 6 summarizes the research results. The implementation details of the GNSS-based camera targeting on the ArduRove-based MAD robot are provided in Appendix A of this paper.

## 2. Related Works

SmartGuard is one of the inspection robots most fully described in research papers [14,15,16,17,18,19,20,21]. It was produced with three different navigation systems (magnetic guidance, Global Positioning System Dead Reckoning (GPS-DR), and visual navigation) and deployed on over a dozen substations in China. According to [18], the version with magnetic guidance was the most precise (error within 0.22 cm). However, this navigation method requires 3-4 weeks of preliminary construction work on each substation. Also, it is not flexible enough because each stop should be defined by a Radio Frequency Identification (RFID) marker embedded in the road, which cannot be easily moved in the future. Interestingly, in 2012, including all the associated costs, magnetic guidance was still considered cheaper than a GNSS by the authors of [18]. The GPS-DR version of SmartGuard provided an error of less than 2 cm at most of the stop points during the inspection of the 500 kV substation. At the same time, the error exceeded 80 cm at a few points. The authors of [18] claim that these peak errors were caused by power lines that stop or disturb the GPS signal from the satellites. The omnidirectional vision system used on SmartGuard was initially tested on a Frontier-II robot [22]. It required installing artificial landmarks in a special pattern, each 2 m on both sides of the road. This method provides a positioning error of ±4 cm in distance and ±2.5° in angle and may work in daylight and night conditions. Unfortunately, Refs. [18,22] do not provide any direct information on the heading accuracy of other SmartGuard navigation systems. Still, the authors of [20] state that a robot navigated by using magnetic guidance may have significant camera targeting error compared to the field of view angle, especially when the camera has a large focal length. To solve this problem, Ref. [20] proposes to use an image-based visual servo control system that compares the camera view with the corresponding template image, evaluates the offsets, and adjusts the camera orientation. It is worth mentioning that such servo control systems have significant limits in terms of the light and weather conditions and may incorrectly operate in cases of equipment appearance changes due to aging or damage.

Summarizing the experience of SmartGuard, it shows the feasibility of the GNSS-based navigation on the 500 kV substation under voltage. At the same time, information on the actual accuracy and the other characteristics of this navigation method provided in the published papers is limited. Moreover, it can be considered outdated due to the modern GNSS receiver and sensor fusion systems providing more robust satellite reception at lower prices than those used in the 2010s.

Laser positioning is another common navigation approach in many application areas, including power plant and substation inspection. It can rely on a system of reflectors with predefined positions [23] or operate without them using so-called LiDAR odometry [4]. Reflector-based laser positioning can provide lateral navigation errors of ±15 cm and heading deviations of ±1.5° [23]. Ref. [24] describes the complex multi-sensor system, including a 3D LiDAR, IMU, and computer vision, installed on a robotic dog, which provides, during the operation on a substation, a distance error around 1 m and an angular error of approximately 10°. At the same time [4], an approach with much better precision regarding LiDAR odometry claimed that the extended Kalman filter (EKF) covariance used in their all-weather navigation system was 0.1 m for translation and 1° for orientation angles. This result is much closer to the state-of-the-art results achieved in other application areas outside power equipment inspection [25,26]. Heading estimation is one of the weakest points of laser positioning. Even with stationary reflectors used as landmarks, the final mapping results include angular distortion exceeding 1.5° [27]. The distortion was estimated by the authors of this research from the Figures 9a and 10a published in [27]. Without the reflectors, the 2D LiDAR SLAM may lead to robot course error up to ±3° and even higher [25]. Such angular errors are generally not critical for robot navigation on the route but significantly complicate the targeting of narrow-angle cameras, especially if the regions of interest were chosen using substation models but not in the field. In the last case, due to the angular distortions, the inspection region of interest may have different coordinates on the LiDAR-generated map compared to the geo-referenced model.

Another possible technology to provide navigation for the inspection robot is UWB. It is usually used to provide navigation indoors (e.g., underground substations [28]) to replace the absence of a GNSS but can also be used outdoors [29,30] to improve the navigation performance when GNSS signals are temporarily unavailable or have low quality. The positioning error standard deviation of the UWB systems tested on electrical facilities by different algorithmic approaches and fusion with an IMU can be lowered to a 5 cm level. Similarly, the heading estimation deviation can be reduced to ±2.5° [28,29,30]. These results are similar to the ones achieved in the other application areas [31,32,33]. One of the best results was reported by [33], providing positioning and heading errors of about 4 cm and ±1.3°, respectively. Interestingly, the last result was verified by GNSS RTK systems used as a reference and considered more precise.

From the application task point of view, all the navigation methods described above provide enough accuracy for positioning the robot along the inspection route but may be insufficient to target a narrow-angle camera. For example, the error of the laser positioning system within 10–15 cm accuracy is much lower than the distance between the robot and the regions of interest, the average road width, and even the dimensions of the robots. At the same time, the influence of the heading estimation error on the camera targeting is much more significant because it is directly added to the camera targeting error along the yaw axis. In the case of a narrow-angle inspection camera with an 8° FOV, a heading error of ±2.5° will lead to losses of up to 31% of the power equipment image.

Generally, a GNSS RTK is designed to provide only the rover’s position. Meanwhile, two GNSS receivers installed on one robot can be configured into a moving baseline RTK and used to estimate the heading. The precision of this method highly depends on the distance between the GNSS antennas. On short distances below 0.5 m headings, the standard deviation may exceed 1°. However, if the baseline length is equal to 1 m or longer, the error becomes lower than 0.3° [34], outperforming all the other heading estimation methods used on inspection robots. The last statement is indirectly confirmed in [4], where the EKF covariance of the dual-antenna RTK GNSS heading is much lower than the other sensors used in the inspection robot’s navigation systems. Unfortunately, Ref. [4] does not provide any additional information about the so-called GNSS compass performance in electrical substation conditions that can be considered while designing the other robots. Moreover, from the provided data, it is not clear if the other navigation sources of the robot (LiDAR and wheel odometry) were obligated to reach the experimental studies’ results or if the inspection robot could also achieve them by relying on the GNSS and IMU only for both position and heading estimation. Instead, the authors state that the other sensors are needed because buildings and power transformers can interfere with GNSS satellite signals and lead to unreliable GNSS localization.

The risk of bad GNSS reception quality is one of the most common reasons to use other navigation approaches. At the same time, it may not be as critical for the inspection applications as it seems. The data acquisition with narrow-angle cameras generally assumes a significant distance (20–30 m) between the robot and the region of interest. In this case, the keypoint from which the robot targets its camera can usually be moved within several meters to provide the best GNSS quality and avoid standing near large objects fully covering the sky. Moreover, the GNSS reception quality can be easily estimated by such parameters as the number of satellites providing navigation data, the horizontal and vertical dilution of precision (HDOP and VDOP), and the GNSS RTK status (RTK fix, RTK float, etc.). Thus, keypoints with bad GNSS reception quality can be easily determined and corrected.

To summarize the analysis of the related works, the GNSS RTK is the most precise navigation approach for inspection robots regarding position and heading estimation besides magnetic guidance (magnetic guidance is excluded from consideration as it requires construction work to change the inspection route). Due to its high precision, it is often used by researchers as a reference to estimate the performance of other navigation sensors such as LiDARs, IMU, UWB, cameras, and others [33,35,36,37]. Fusion with IMU data is a common way to improve the positioning accuracy of a navigation system, regardless of its type. The feasibility of GNSS/IMU navigation for electrical facilities was proven in the 2010s. At the same time, most of the available data regarding its actual precision, reliability, and cost of implementation are currently outdated. Finally, the recent papers on the topic, like [4], do not provide engineers with enough numerical data to make a well-founded decision about whether a GNSS RTK can be used as the sole source of navigation data or whether the inspection robot should be equipped with other sensors.

## 3. MAD Robot and Experimental Sites

### 3.1. Multi-Spectral Automatic Diagnostic (MAD) Robot

**Figure 3:**
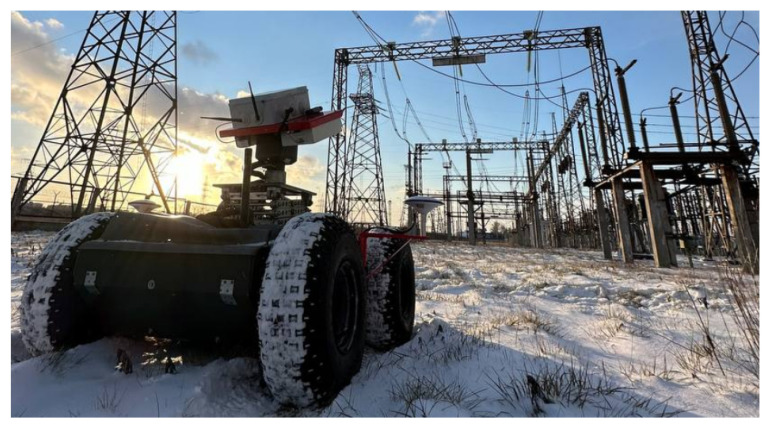
MAD robot during the inspection in one of the experimental sites.

All the experimental studies in the current research were conducted using the MAD robot, which is described in detail in [6,7]. This robot (Figure 3) has outer dimensions of 1500×1300×1100 mm (width × length × height). It is driven by four 500 W electrical motors, which accelerate it up to 3.5 m/s. Nevertheless, the cruise speed used during autonomous inspection routes is 1.3 m/s. The robot has two cameras installed on the pan-and-tilt rotation device: an Ofil Rail HD UV camera (OFIL Systems, Ness Ziona, Israel) and GIT UR-640M (KARNEEV SYSTEMS, Moscow, Russia) combined infrared/RGB camera. The UV camera has a smaller FOV. Thus, this camera will be further used in the current research to estimate the targeting repeatability.

The navigation system of the MAD robot combines an IMU and two GNSS RTK receivers built on low-cost Ublox F9P (Ublox, Thalwil, Switzerland) chips. The same GNSS receiver is used on a static base to provide an RTK correction to the robot, which is transmitted to the robot through a radio modem. The overall cost of all the GNSS RTK navigation systems (including the antennas and the navigation controller that performs sensor fusion but excluding the radio modem) is around 2200 USD (the estimate is based on Aliexpress prices and is valid at the end of December 2023), which is generally less than several weeks of construction work required to install the RFID markers or reflective landmarks on a large substation.

**Figure 4:**
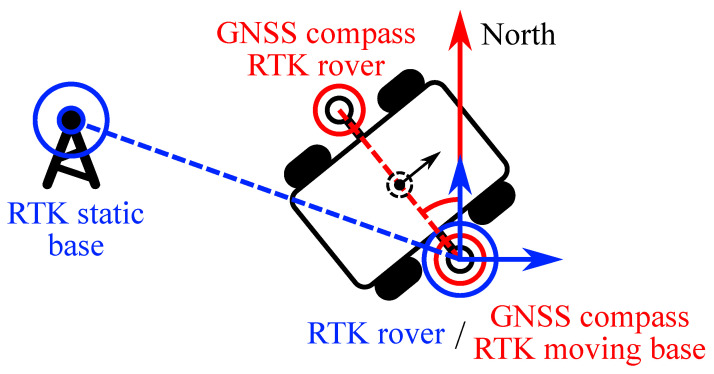
GNSS RTK structure of the MAD robot.

The robot’s navigation system includes an RTK system (Figure 4). The first one combines two receivers installed on the mobile platform. It is configured as a moving baseline RTK and acts as a GNSS compass to estimate the heading. The distance between the RTK antennas on the robot is 1.24 m. The second GNSS RTK is used to determine the robot’s position precisely. It combines the right side antenna, installed on the robot, and the static base in the point with the location pre-measured during a geodetic survey. The navigation computer fuses both RTK systems with the IMU and estimates the heading and center of mass position.

### 3.2. Experimental Sites

The experiments were performed at three different sites. The first site (site 1) is a field crossed by the overhead power lines located in the Moscow region. It is characterized by the fully open sky and the absence of buildings or metal constructions that may influence GNSS signals. The second site (site 2) is a 220 kV substation of a 400 MW power plant located in the European part of the Russian Federation. The power plant and the substation are built on a lower part of a river’s steep bank in a relatively small area for such a type of facility. As a result, all the equipment is placed as close together as possible according to the safety rules. Moreover, the GNSS signal quality is potentially influenced not only by the power equipment and buildings but also by a steep slope. The third site (site 3) is a 220 kV substation of a 2400 MW power plant located in the Asian part of Russia. It is built in an open plain, so the terrain does not affect the reception of the GNSS signals. At the same time, the power plant is much larger than the one at site 2.

The experimental studies at the first two sites were performed during single inspection visits for each of them during the summer of 2023. Contrarily, the substation at site 3 was inspected multiple times in summer, autumn, and winter, which made it possible to analyze the long-term deviations in the GNSS precision and repeatability.

All three experimental sites have distinctive features. Site 1 provides near-ideal GNSS reception conditions typical for the overhead power lines inspection. At site 2, the GNSS antennas can be partially shaded by the terrain or closely aligned equipment. Even though the energy generated by the plant at site 2 is much lower than that of site 3, its substation is an important transfer node in the regional grid. Finally, the substation at site 3 is located in an open area, but it is connected to a 2400 MW power plant and provides energy to multiple large industrial facilities. Finally, the spatial distribution of all three sites leads to the reception of different sets of satellites and makes the experimental results more representative.

## 4. GNSS Reception Quality and Positioning Accuracy

### 4.1. GNSS Reception Quality

**Figure 5:**
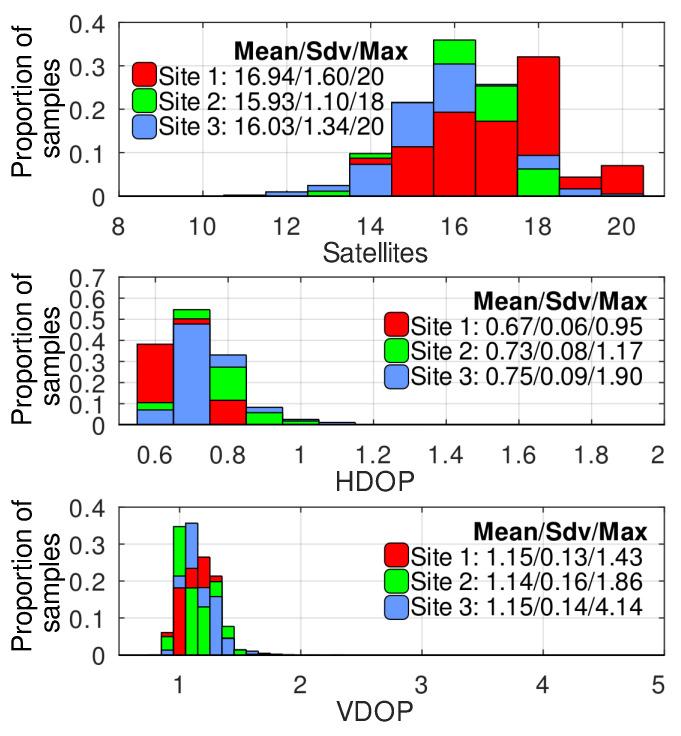
The histograms of satellite count, HDOP, and VDOP distributions at different experimental sites.

The GNSS reception quality was evaluated by analyzing the number of satellites providing the navigation data, horizontal and vertical dilution of precision (HDOP and VDOP), and GNSS RTK status during the inspection routes. The data from the inspections at all three sites are presented as histograms in Figure 5 (here and below, the histograms from the different sites are overlapped so that the lower bars are plotted above the higher ones. Additionally, statistical parameters, such as mean value, standard deviation (Sdv), and a maximum of absolute value (Max), are presented in a legend). The histograms presented in Figure 5 are built using data from three different inspection sites representing the conditions typical of power equipment monitoring. These data were collected during automatic inspections and covered both the keypoints from which the diagnostic information was recorded and the ones passed by a robot while following the routes. The significant parts of the missions at sites 2 and 3 passed below the 220 kV conductors located at an average height of 7.2 m from the ground.

As observed, the average number (rounded to the closest integer) of the satellites simultaneously providing navigation data for the robot is 16. This number is more than twice the minimum (seven satellites) required by the Ublox receiver to switch into the RTK Fix state. The HDOP, most of the time, is below 1, which is rated as ideal reception conditions according to the classification provided in [38]. Moreover, it never exceeds 2, the border of an excellent rating. The VDOP parameter was generally higher than the HDOP at all the sites. However, most of the time, it was in the range corresponding to excellent conditions, and, only in a few short periods at site 3, it dropped to a good level. Thus, it is unsurprising that the proportion of time the robot’s GNSS receivers spent in RTK Fix during the inspection missions was very high. At sites 1, 2, and 3, it was, respectively, 100%, 99.94%, and 99.37%.

It is worth noting that there were several cases when the GNSS receiver went into a 3D Fix state at sites 2 and 3. A detailed analysis showed that, in most of these cases, the decrease in the positioning accuracy was caused by the loss of the RTK corrections transmitted on the robot by the RTK base station. In some cases, it was due to radio communication malfunctions; in others, it was caused by the disconnection of the wires between the RTK base receiver and the radio modem due to human error. Also, at experimental site 3, there were two cases of the receiver switching to 3D Fix mode without the possibility of achieving RTK Fix even in perfect conditions of clear sky and available corrections from the base station. In the first case, the problem was solved by the power sequence. In the second case, the receiver’s functionality was restored only after updating its firmware using the Ublox u-center software v.22.07. Thus, it was concluded that the receiver’s firmware malfunction caused the constant 3D Fix status. Since all the above-described transitions to the 3D Fix state were caused by known factors independent of the GNSS signals’ reception quality or any conditions specific to power equipment inspection, the data collected in the 3D Fix state were excluded from further analysis.

Many papers (e.g., [4,15]) present qualitative evaluations of the GNSS reception conditions at power plants and substations. As a contribution of this research, we provide the quantitative results collected at three different experimental sites, making it possible to compare the navigation conditions with the other applications where the GNSS RTK is more widespread. Thus, the direct comparison with [10] shows that the conditions typical for power equipment inspection are similar to or even better in terms of the satellite number and dilution of precision than the ones evaluated in the vineyard. There is often an opinion among power engineers that, at the substations, due to electromagnetic interference, there are unique conditions that dramatically affect the quality of the satellite signal reception. Our results refute this misconception.

### 4.2. GNSS Accuracy

The GNSS accuracy was estimated by recording the position deviations at each keypoint while the diagnostic equipment collected the data. The MAD robot wheels were blocked during the data acquisition. Thus, all the changes in the robot position according to its Ublox F9P receivers can be considered GNSS RTK deviations. It should be noted that these deviations were recorded directly from the receiver before the sensor fusion with the IMU.

**Figure 6:**
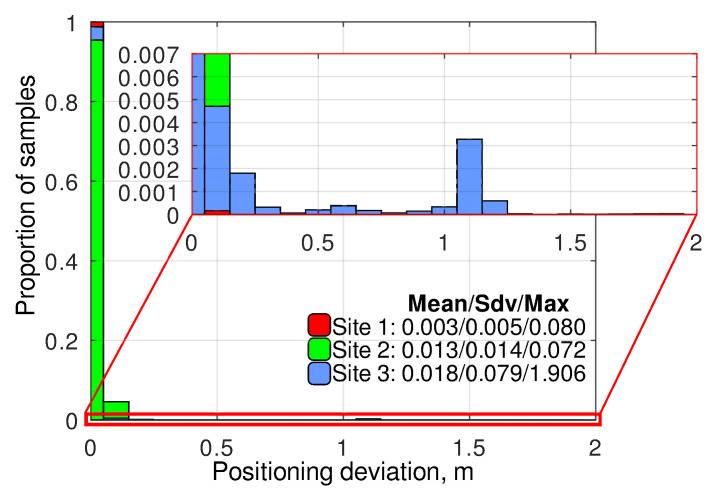
The histograms of GNSS RTK position deviation distributions recorded at different experimental sites at the inspection missions’ keypoints.

The experimental results (Figure 6) show that the position deviation is below 0.1 m in most of the cases. At the same time, in very rare cases, the GNSS accuracy can degrade up to 2 m. All such cases were registered at site 3. Still, it can be described that the experimental studies at this site were performed during several visits and were much longer than at the other sites. Short peaks of position deviation generally do not affect the robot movement, thanks to the fusion with the IMU, but they can influence the inspection results in those cases in which the camera pitch and roll are evaluated once before targeting (it is a common solution to avoid undesired camera movement during diagnostic data acquisition).

**Figure 7:**
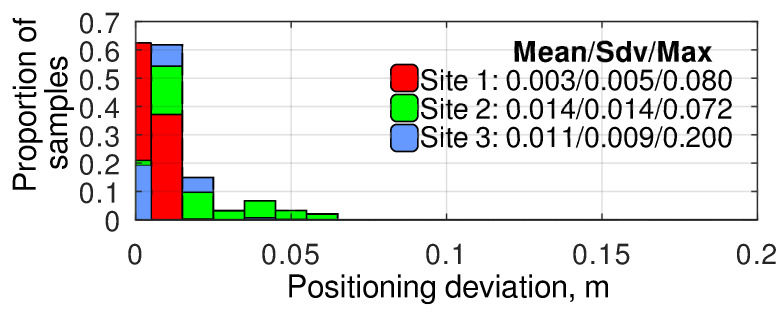
The histograms of GNSS RTK position deviation distributions recorded at different experimental sites at the inspection missions’ keypoints, excluding the measurements that were provided by the GNSS receiver in the RTK Float state.

The close analysis of the measurements with the position deviation above 0.5 m showed that all of them were collected when the GNSS receiver was in RTK Float state. If we exclude those points, the deviations will decrease to the levels typical for the GNSS RTK in excellent reception conditions (Figure 7). Thus, it can be concluded that the RTK state is a reliable indicator of high GNSS accuracy, and the estimation of pitch and yaw angles used for camera targeting must be performed using only the coordinates obtained in the RTK Fix state. Moreover, it was evaluated that, in all the cases, when the receiver switched to the RTK Float mode, it took no more than 1.2 s to switch back and provide a precise position. As a result, it is possible to wait for the RTK Fix state after the robot reaches each keypoint without a significant increase in the inspection duration. The details regarding how such a delay can be implemented for an ArduRover-based MAD robot are provided in Appendix A.

### 4.3. Robot Positioning Repeatability

**Figure 8:**
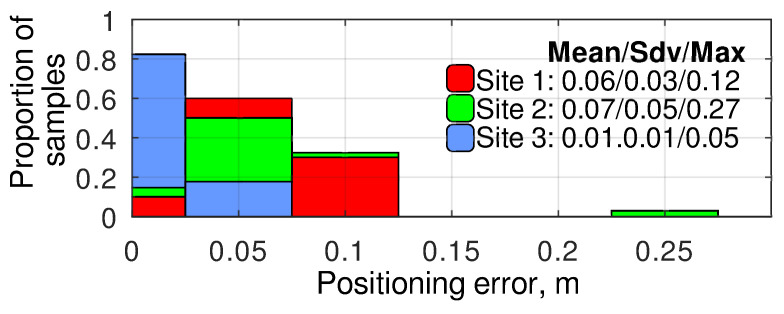
Histograms of positioning error recorded at different experimental sites at the inspection missions’ keypoints.

The robot positioning repeatability was measured by comparing the position on the sensor fusion output with the IMU at the same keypoints reached during different iterations of the inspection missions. The compared position was estimated at the moment the robot stopped. The experimental results (Figure 8) show that the positioning repeatability, which depends on many factors besides navigation (e.g., terrain conditions), is better than 0.1 m in most of the cases. While analyzing these results, it should be considered that they are obtained using the internal navigation system of the robot, which is affected by the RTK GNSS position deviations discussed in the previous section (Figure 6). However, even considering these deviations, the resulting positioning accuracy is sufficient to ensure the repeatability of the camera angles when repeating the same routes multiple times. This repeatability is essential for preparing datasets for artificial intelligence and machine learning methods [7].

## 5. GNSS Camera Targeting Repeatability

### 5.1. Camera Targeting Methods

**Figure 9:**
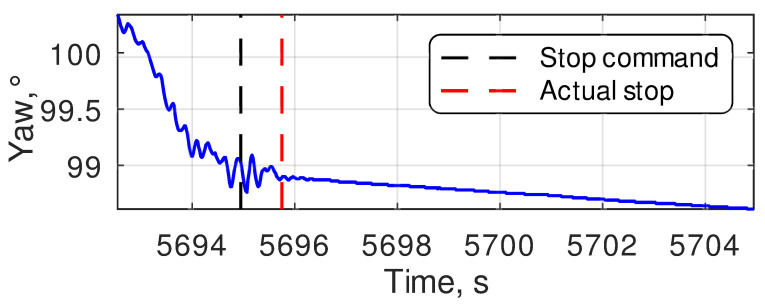
An example of yaw estimation transient process at the moment of stop at a keypoint.

**Figure 10:**
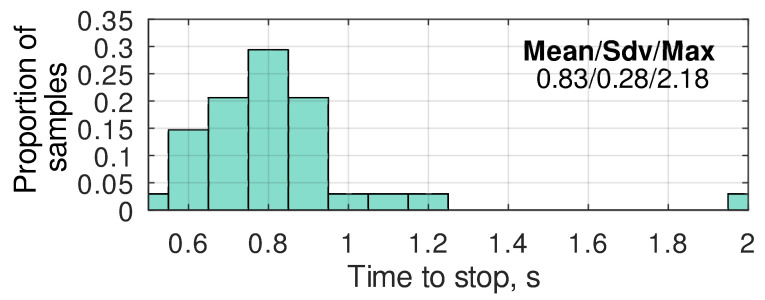
The histogram of the time required to settle down yaw estimations after a stop at a keypoint.

The robot’s navigation system used in the current research is based on the ArduRover firmware [6,7]. Its default camera targeting procedure is implemented using the DO_SET_ROI command, which automatically evaluates the camera pitch and yaw angles based on the current robot position and orientation. Typically, these angles are cyclically updated, causing permanent image jitter in narrow-angle cameras due to sensor noise. To avoid this, the motion controller of the robot reads out the pitch and yaw setpoints a single time before targeting and does not change them until the diagnostic equipment finishes collecting the data. Such behavior solves the problem of camera jitter. However, it raises the requirements for the quality of the position at the moment when the setpoints of the pan-and-tilt rotation device are read. The camera targeting should be started only in the case that both of the GNSS RTK receivers are in the RTK Fix state to provide such quality. This is the most important, but not the only, requirement. The noise in the yaw estimation dramatically affects the quality of the targeting. After stopping at the keypoint, it was noticed that the yaw value needs some time to settle down (Figure 9). A detailed analysis of such behavior showed that it was caused by the vibration during the stop. By default, ArduRover firmware switches to the next part of the mission when it sends a stop command (zero velocity setpoints) to the motion controller. At the same time, due to the two relatively high masses, blocking the wheels causes high vibrations on the chassis and antenna holders, resulting in temporary yaw inaccuracy. As measured, the time between the stop command and actual stop in most cases is below 1 s (Figure 10), and the maximum delay registered during the experimental studies was 2.18. Thus, as a compromise between the accuracy of the angle estimate and the increase in the inspection duration, a delay of 1.5 s was added to the mission program after each stop at a keypoint. The targeting procedure described above is a standard one provided by ArduRover firmware. Further, in this paper, it will be called the ROI method. The targeting precision of this method directly depends on the precision of the GNSS compass heading estimation.

During the initial stages of the current research, it was noticed that, at keypoints from where multiple parts of the equipment should be shot, the targeting accuracy of the first shots was higher than the further accuracy. We assumed that, when the robot is at a standstill for a long period, the yaw drift on the output of the sensor fusion procedure causes this inaccuracy (the time required to collect the diagnostic data for one part of the power equipment varies from 30 s to 1 min). This drift can be observed on the right side of Figure 9. When the robot moves, its yaw estimation is corrected using the speed vector direction. Still, when it stops, the noise of the IMU sensor results in yaw inaccuracy, which is bounded by the GNSS compass precision. The attitude drift during the robot standstill is a known issue of the GNSS/IMU navigation system, which is usually suppressed using the Zero Velocity Update (ZUPT) method [39]. Unfortunately, ArduRover does not support ZUPT yet, and one of the aims of our project was to implement the navigation system without custom firmware. As a workaround, we propose a relative targeting method that can be implemented using the currently available ArduRover build. The specific implementation details related to this targeting method are provided in Appendix A.

The new approach assumes that the best yaw estimation quality at the keypoint is reached just after the robot stops. According to the relative targeting method, the robot targets the first part of the equipment ($r_{0}$) the same way as in the ROI method. The corresponding yaw $\varphi_{0}$ and pitch $\alpha_{0}$ setpoints are stored in the memory of the robot’s motion controller. For the rest of *N* parts of the equipment ($r_{0}$…$r_{n}$), the setpoints of the pan-and-tilt rotation device are evaluated using (1) and (2).

(1) $$\varphi_{r_{n}}=\varphi_{0}+\varphi_{n},$$

(2) $$\alpha_{r_{n}}=\alpha_{0}+\alpha_{n},$$

where *n* is the index of the power equipment part in the inspection sequence performed from the keypoint; $\varphi_{r_{n}}$, $\alpha_{r_{n}}$ are the setpoints of the pan-and-tilt rotation device used to target the camera on the $r_{n}$ equipment part, and $\varphi_{n}$ and $\alpha_{n}$ are the yaw and pitch angles between the directions on the $r_{n}$ and $r_{0}$ equipment parts, which were pre-evaluated during the mission planning using keypoint coordinates.

**Figure 11:**
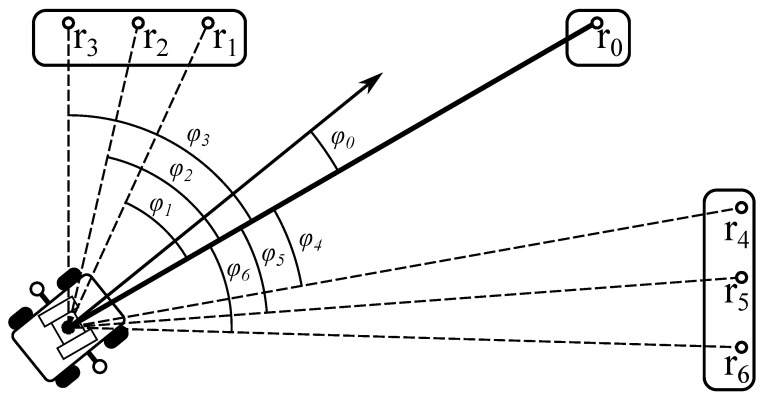
An illustration of the camera yaw targeting using the relative targeting method.

Figure 11 illustrates how the yaw angles are defined in the relative targeting method (the pitch angles are determined similarly).

The relative targeting method is designed to increase the camera targeting repeatability when many equipment parts are inspected from a single keypoint. Its operating principle is based on two assumptions: (1) the robot’s positioning repeatability is high enough so that the difference between the actual robot position and keypoint coordinates can be considered insignificant; (2) the pan-and-tilt rotation device is equipped with a precise encoder, which guarantees providing the targeting error far below the precision of the GNSS compass.

The targeting error introduced by the mobile platform’s positioning error can be estimated using (3).

(3) $$\varepsilon =\arcsin\frac{\delta }{d},$$

where δ is the mobile platform’s positioning error, and *d* is the distance from the keypoint to the inspected part of the equipment.

During the current research, in most of the cases, the parameters of (3) were δ≤0.1 m (Figure 8) and d≈30 m, which results in a maximum error ε of 0.19°. The encoders of the pan-and-tilt rotation device of the MAD robot used in the current research provided positioning accuracy of at least 0.018°. According to the Ublox F9P receiver datasheet, the heading estimation accuracy using a moving baseline with length 1.24 m is approximately 0.36°. Thus, the relative targeting method can potentially provide approximately 1.73 better targeting accuracy than the ROI method for the routes where many camera angles are shot from a single point. The precision boost can be even more significant in practice because (3) represents a worst-case scenario.

The details of the relative targeting method implementation on the ArduRover-based MAD robot are presented in Appendix A. Our experimental studies also discovered two non-obvious drawbacks of this method. First, the relative angles encoded in mission commands generally lie in a twice-larger range than camera yaw and pitch setpoints. In the robot used in the current research, these angles were transmitted to the motion controller through the S.Bus interface [6], which uses only 11 bit channels. Thus, to cover the range of the relative setpoints, their precision should be decreased by two times compared to the absolute setpoints used in the ROI method. As a partial solution in the current research, we used the same precision for both types of setpoints but chose the first inspected part in a way that it is situated in the middle between the other equipment parts that should be inspected from the same keypoint (e.g., Figure 11).

The second discovered drawback is that the relative targeting method requires approximately 1.5× commands to program the mission rather than the ROI method in the current implementation. Currently, the robot’s navigation controller can store up to 1312 commands, enough to fit a 1.5 h mission for inspecting around 300 equipment parts using the ROI method. Using the relative targeting method will reduce the maximal mission length to approximately 1 h and 200 inspected objects.

### 5.2. Targeting Repeatability

We generated a pair of inspection missions to compare the targeting repeatability on each site. These missions were planned using the same set of keypoints and inspected equipment parts but with different targeting methods. Then, each mission was executed multiple times. In all the keypoints, the targeting procedure started only when both GNSS receivers of the robot were in the RTK Fix state.

**Figure 12:**
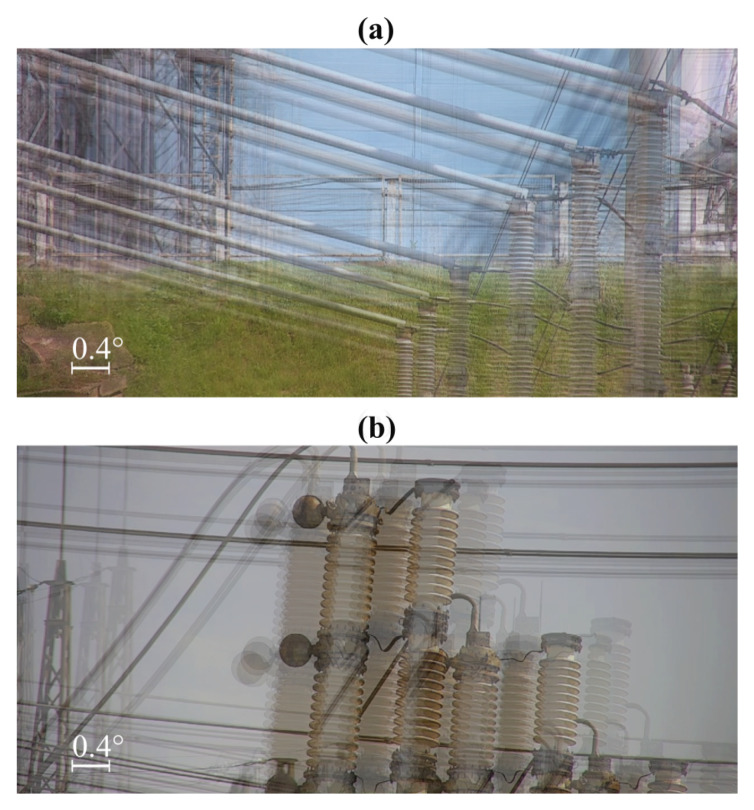
Examples of frame sets used to estimate the camera’s repeatability at experimental sites 2 (**a**) and 3 (**b**).

The inspection results collected on each site were sorted by the inspected equipment part. Thus, each part corresponded to several UV videos obtained during different iterations of the same inspection mission. Generally, this video contained close to a static RGB background overlayed with corona discharge zones. Then, from each video, a frame with a minimal corona discharge zone area was chosen as the keyframe. All the keyframes corresponding to the same inspected part were matched with the one collected during the first iteration of the mission. The match was performed using Speeded-Up Robust Features (SURF) [40] and resulted in the scale, rotation, and shift of each key frame relative to the first one. The first keyframe used as a reference during the matching procedure was influenced by the same sources of error as the other keyframes; the mean value of the vertical and horizontal shifts among all the frames related to the same equipment part was subtracted from the corresponding shifts of each keyframe. Finally, the shifts (estimated in pixels) were converted to vertical and horizontal angular errors using the known FOV of the Ofil RailHD camera (8°×4°). Figure 12 shows examples of the frame sets used to estimate the camera’s repeatability at various keypoints. All the frames in each set are overlaid for clarity.

**Figure 13:**
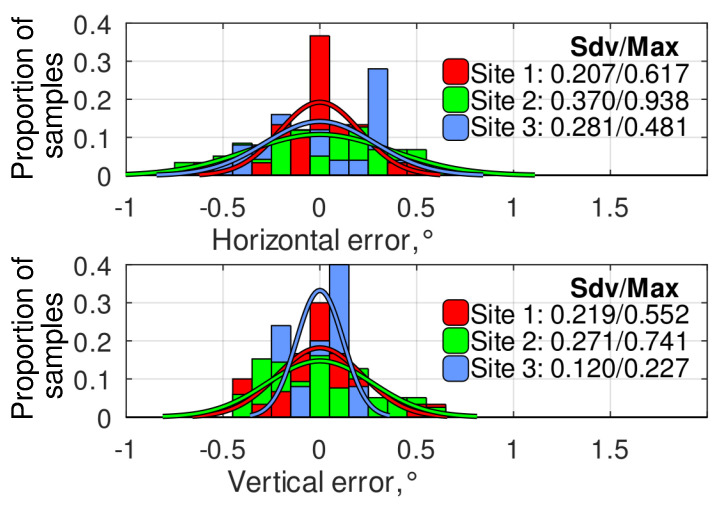
The histograms of angular error distribution at different experimental sites using ROI method.

**Figure 14:**
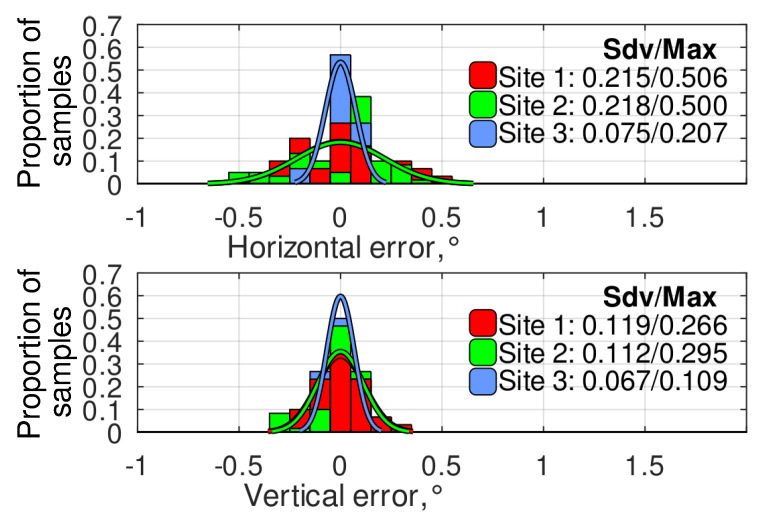
The histograms of angular error distribution at different experimental sites using the relative targeting method.

The histograms of the angular error distribution at different experimental sites using the ROI and the relative targeting methods are presented in Figure 13 and Figure 14, respectively. As observed, the relative targeting method generally provides a lower standard deviation and up to 2.5 times lower absolute maximum error than the ROI method, solving the problem of the navigation error accumulation while the robot is at a standstill. It can be noticed that, at site 3, the relative targeting method provided much better targeting accuracy than the two other experimental sites. It is explained by the lower positioning error at this site (Figure 8), which is highly important for the relative targeting method as the relative setpoints are evaluated during the mission generation and do not depend on the actual robot’s position. Finally, it can be concluded that a navigation system based on modern low-cost GNSS RTK receivers can provide camera targeting with accuracy within ±1°. At the same time, the implementation of the relative targeting method proposed in this paper can bind the error to ±0.5°.

### 5.3. Comparison with Other Navigation Approaches

The area of experimental site 3 was 3D-scanned as a part of a more complex industrial project. The scanning was performed using EFT SL1. This scanner is equipped with precision 3D LiDAR. It uses SLAM to estimate and combine the measurements obtained from different points in real time. EFT SL1 costs around 46000 USDthe estimate is based on the price on an official web portal of the EFT Group and is valid at the end of December 2023) and is superior to many of the LiDAR SLAM navigation systems designed for mobile robots. The scanner’s precision declared by the manufacturer is 0.1 m, which makes it comparable to the navigation system of the robot used in the current research.

**Figure 15:**
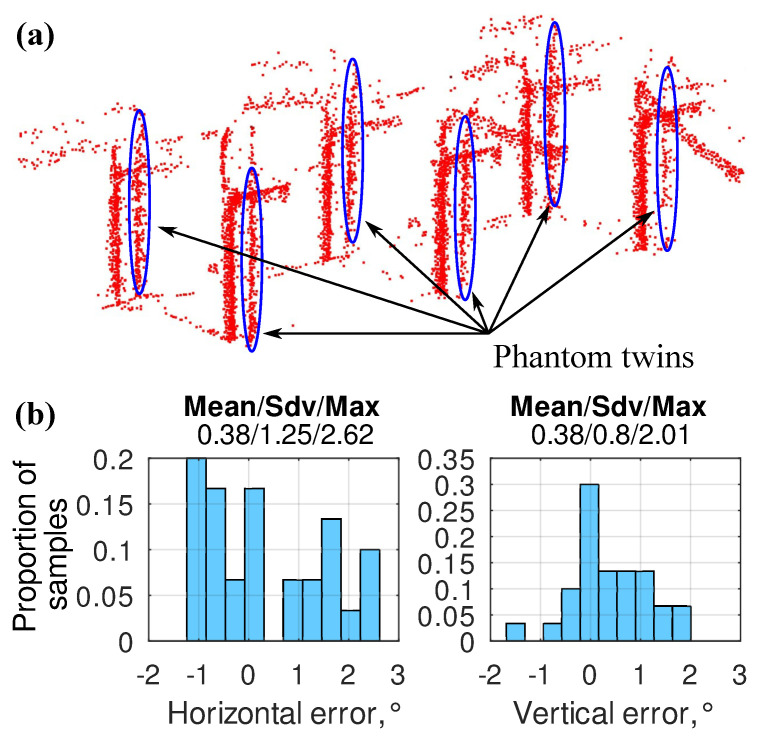
(**a**) An example of phantom twins (marked by blue ovals) resulting from a LiDAR SLAM error. (**b**) The histograms of LiDAR SLAM camera targeting errors estimated based on phantom twins position on the substation’s 3D model (site 3).

The analysis of the 3D scan showed that some parts of the power equipment have phantom twins (Figure 15a). These twins appear when the SLAM algorithm does not correctly merge different point clouds. Such errors often occur in the parts of the substation where there is little or no equipment and the LiDAR does not provide enough points for SLAM. A similar problem was described in [27], so it can be considered typical for LiDAR SLAM. Nevertheless, the angle between the part of the equipment and its phantom twin measured from the keypoint from which this part of the equipment should be inspected can be used as an indirect estimation of the camera targeting error in the case that the MAD robot was navigated using LiDAR SLAM. The histograms built based on these angles are presented in Figure 15b.

It should be noted that the histograms do not fully represent the LiDAR SLAM error distribution because they do not include situations when SLAM worked correctly and the phantom twin did not occur. At the same time, Figure 15b is valid for evaluating maximum targeting errors. It shows that, at site 3, these errors of LiDAR SLAM were 12–18 times higher than the ones provided by the GNSS RTK targeting.

A comparison of the targeting error achieved with different navigation systems is summarized in Table 1. Since most papers have no direct information on the targeting error in such cases, it was estimated as a sum of the heading error ε and evaluated according to (3). For all the estimations, *d* was assumed to be 30 m, and δ was equal to the positioning error reported in a corresponding paper. The results of [8] are included in Table 1 for targeting without Active Pose Relocalization (APR) because APR serves as an independent extension that can be applied to any system listed in Table 1.

As observed from Table 1, GNSS RTK/IMU navigation is not only suitable for narrow-angle camera targets but also significantly outperforms the other navigation approaches, especially when it is used with targeting following the relative targeting method proposed in the current paper.

The angular discreteness of the outgoing laser ray explains the high targeting error of the LiDAR-based methods. The equipment in power plants and substations is usually not solid, and most LiDAR rays go through instead of reflecting from narrow metal frames. As a result, the point clouds processed by SLAM algorithms do not have enough density, leading to a rise in the heading estimation error, which is directly included in the targeting error. The other methods based on laser emissions suffer from the same problem, especially when the robot passes the open areas inside a substation [27]. The UWB and GNSS RTK navigation systems can directly estimate the robot’s heading regardless of the density of the surrounding equipment. At the same time, GNSS RTK systems are currently more precise than UWB ones. Moreover, a GNSS is generally less influenced by non-line-of-sight signal propagation problems because the wires and conductors above the robot shadow much less of the sky than the equipment that shadows the line-of-sight path to the UWB beacons installed on the substation.

The experimental results show that the GNSS RTK can be considered a self-sufficient technology for MAD robots. Its fusion with the IMU is a well-known approach to minimizing the influence of satellite signal quality variations. At the same time, the GNSS RTK fusion with other navigation technologies, such as LiDARs [4], seems to be excessive for power equipment inspection applications, especially in terms of cost-effectiveness.

**Figure 16:**
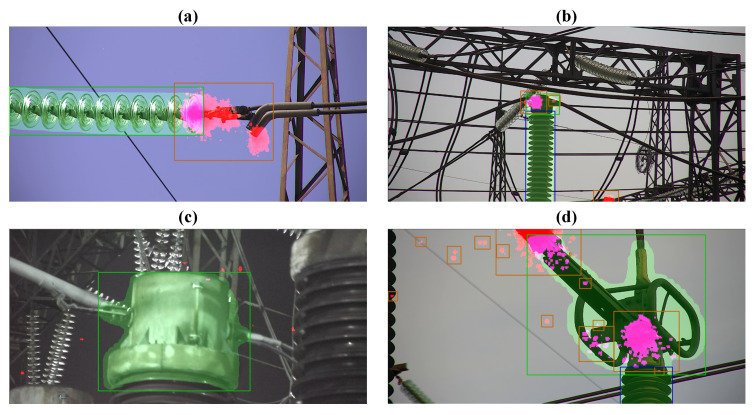
The examples of UV images captured in different conditions: (**a**) an insulator string, summer, sunny day; (**b**) post-insulator, autumn, cloudy day; (**c**) current transformer, winter night; (**d**) disconnector, spring, cloudy day. Red zones—corona discharge activity registered by UV camera. Green zones—the equipment detected by the inspection software.

These statements are supported by real inspection missions performed using the MAD robot, confirming that our approach provides robust navigation and camera targeting in all seasons, both in daylight and at night. Figure 16 demonstrates examples of UV images captured in different conditions. As observed in all the cases, the captured equipment is located in the center of the frame and successfully identified by the inspection software (the inspection software was developed by the members of the research team and will be described in detail in our future papers), which confirms the high efficiency of the proposed targeting procedure.

It should be mentioned explicitly that, different to the other approaches, the proposed GNSS targeting is not influenced by the lighting conditions (like APR at night) or weather conditions (like LiDARs in snow or rain). At the same time, additional measures may be required to ensure the proper operation of the inspection equipment, which is caused by its limitations. For example, during night missions, we installed a spotlight on the pan and tilt platform of the MAD robot. Also, on sunny days, we replanned the missions, choosing the set of inspected equipment in such a way that the infrared (IR) and UV cameras never had to capture images in the direction of the sun. The last aspect was easy to implement due to the automatic mission generation approach proposed in [7].

## 6. Conclusions

The experimental studies carried out during the current research provided quantitative results representing the GNSS RTK operating conditions during the inspection of power facilities, which made it possible to compare these conditions with the other application areas where the GNSS RTK is more widely used (e.g., agricultural robotics). These results demonstrate the falsity of the statement that substations have unique conditions due to electromagnetic interference that dramatically affect the quality of the satellite signal reception. On the contrary, our measurements at two different substations show that the GNSS quality in terms of the satellite number and dilution of precision is similar to or even better than in vineyards, where the electromagnetic interference is minimal. Moreover, we provided direct measurements of angular errors that could be achieved on operating power plants using GNSS-only camera targeting, which can serve as a reference for other researchers and developers of inspection robots for energy applications.

As a result of the analysis of the factors affecting the MAD robot’s GNSS-based navigation system during the power equipment inspection, we proposed an approach regarding how to reach targeting accuracy below ±0.5° using open-source ArduRover firmware and a pair of low-cost Ublox F9P GNSS receivers. The implementation details are provided in Appendix A. The proposed solution is very cost-effective and outperforms other navigation approaches in this task, including LiDAR SLAM and UWB, making the necessity of their use in substation and power plant inspection applications questionable.

## Figures and Tables

**Table 1:** Comparison of targeting error achieved with different navigation systems.

Source	Navigation System	Area/Targeting Method	Targeting Error
P.Xiao et al., 2013 [23]	Landmark-based laser navigation	Substation/ Estimation	1.8°
J. Zeng et al., 2015 [29]	UWB	Substation/Estimation	2.6°
S. Jiang et al., 2022 [25]	LiDAR/SLAM/ IMU	Greenhouse/Estimation	3.6°
S. Zheng et al., 2022 [33]	UWB/IMU	Laboratory/Estimation	1.4°
Q. Jiang et al., 2023 [8]	LiDAR/SLAM	Substation/Targeting without APR	3°
This paper	LiDAR/SLAM	Substation/Estimation	2.6°
This paper	GNSS RTK/IMU	Substation/ROI method	0.9°
This paper	GNSS RTK/IMU	Substation/the relative targeting method	0.5°

**Figure A1:**
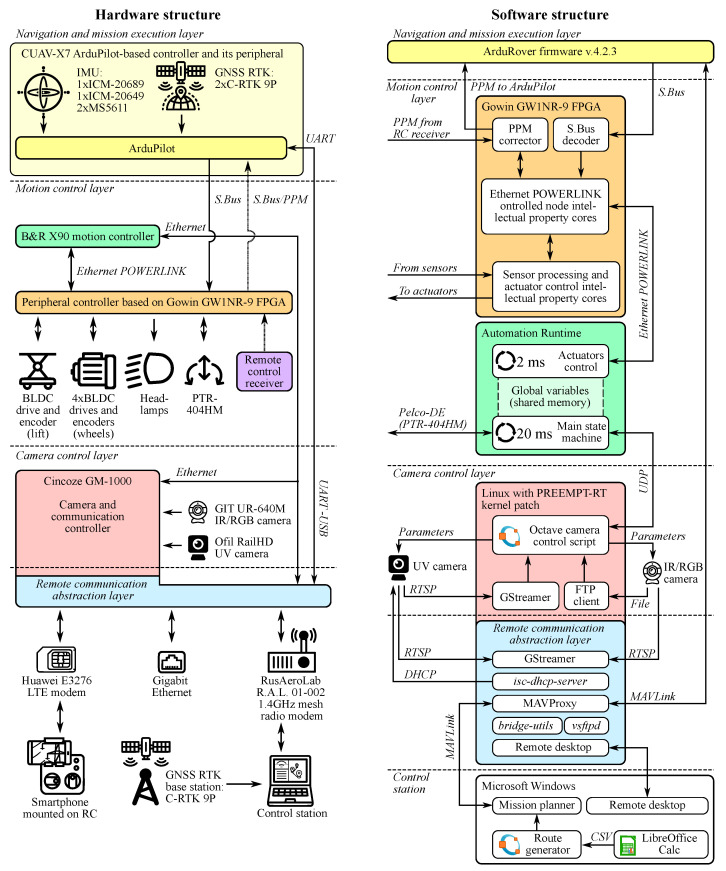
The MAD robot’s control system hardware (left) and software (right) structures [6].

**Figure A2:**
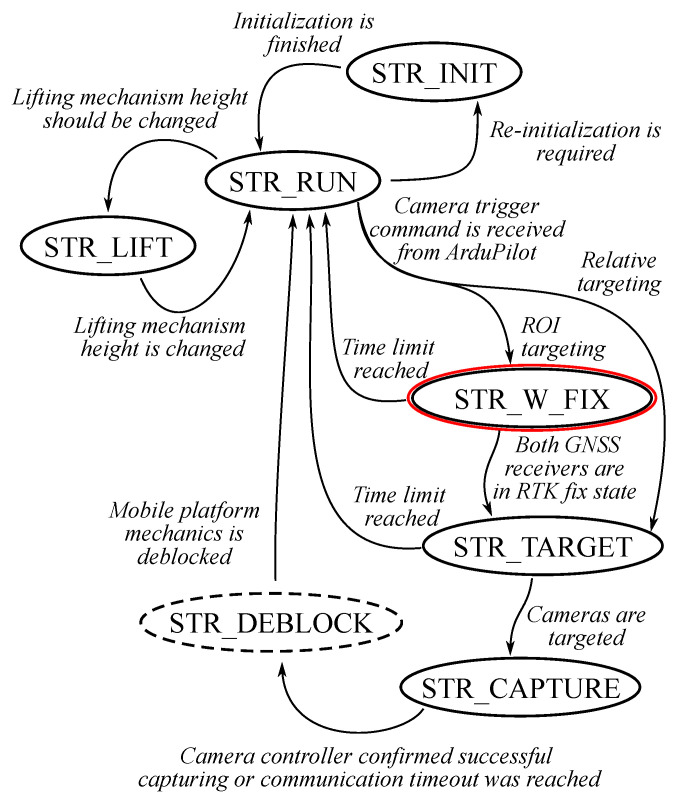
Modified state machine of the MAD robot’s motion controller.

## Data Availability

Data available on request due to restrictions. Due to the fact that the field data were collected at operating power plants, the authors can only provide them in anonymized form, without raw geospatial data. The raw data can be provided upon reasonable request only after obtaining permission from the companies operating the power plants.

## Abbreviations

    The following abbreviations are used in this manuscript:

APR	Active Pose Relocalization
DHCP	Dynamic Host Configuration Protocol
EKF	Extended Kalman filter
FOV	Field Of View
FPGA	Field Programmable Gate Array
FTP	File Transfer Protocol
GNSS	Global Navigation Satellite System
GPS-DR	Global Positioning System Dead Reckoning
HDOP	Horizontal Dilution Of Precision
IMU	Inertial Measurement Unit
IR	Infrared
LiDAR	Light Detection And Ranging sensor
LTE	Long Term Evolution (mobile communication technology)
MAD	Multi-Spectral Automatic Diagnostics
Max	Maximum of absolute value
PPM	Pulse Position Modulation
RC	Remote Control
RFID	Radio Frequency Identification
RGB	Red, Green, and Blue (refers to the visible part of the light spectrum)
RTK	Real-Time Kinematic positioning
RTSP	Real-Time Streaming Protocol
Sdv	Standard deviation
SLAM	Simultaneous Localization And Mapping
SURF	Speeded-Up Robust Features
UART	Universal Asynchronous Receiver/Transmitter
UDP	User Datagram Protocol
USB	Universal Serial Bus
UV	Ultraviolet
UWB	Ultra-Wideband navigation
VDOP	Vertical Dilution Of Precision
ZUPT	Zero Velocity Update

## Appendix A. Implementation of GNSS-Based Camera Targeting on ArduRover-Based MAD Robot

The hardware and software concepts of the MAD robot used in the current research were reported in [6,7]. To implement the relative targeting method proposed in this paper, we made multiple modifications to the software described in [6]. A brief description of the robot’s hardware and software will be provided to make it easier to replicate these changes.

From the hardware point of view, the robot’s control system includes four levels (Figure A1): (1) navigation and mission execution layer; (2) motion control layer; (3) camera control layer, and (4) remote communication abstract layer. Still, only the first two layers are involved in camera targeting. The navigation and mission execution layer is implemented using the CUAV-X7 controller and vanilla version of ArduRover firmware (Figure A1). It communicates with the motion layer through the S.Bus protocol using dedicated channels to transmit setpoints for lift, wheels’ drives, pan and tilt platform, identifiers of the captured equipment, and camera parameters (which are then provided to the camera control layer), as well as a camera trigger signal. We added three more channels to implement the relative targeting method: relative yaw/pitch angles and a servo channel activating/deactivating relative targeting mode. Also, another channel was added to inform the motion controller that both GNSS receivers are connected and have reached RTK fix mode. All the listed above signals were scaled to the 1000..2000 range standard for ArduPilot-based solutions.

The S.Bus protocol is decoded on the motion control layer by a Field Programmable Gate Array (FPGA) and re-transmitted to the B&R motion controller using Ethernet POWERLINK fieldbus. Also, the FPGA-based peripheral controller processes S.Bus/PPM signals from the remote control receiver, making it possible to force ArduPilot to switch into Hold mode during camera targeting and data acquisition procedures (the detailed description is provided in [7]). The key idea of the described control system concept is using ArduPilot to solve all the navigation and mission execution tasks and Mission Planner software to control the robot.

The first modification that was added in the current research compared to the [6,7] is Lua script that checks GNSS receivers’ state and switches Script1servo output into high level (2000) when both of the receivers reach RTK Fix state. The script’s algorithm is provided below (Algorithm A1). The detailed description of how the Lua script can be activated in ArduPilot can be found in [41].

Another modification was made to the motion controller state machine by adding a new STR_W_FIX state (Figure A2). The state machine switches to this state in the ROI targeting method and waits until the GNSS receivers reach the RTK Fix state before switching to the STR_TARGET state. It is worth mentioning that the first part of equipment in each keypoint is always captured using the ROI method, so the STR_W_FIX state is also used to store $\varphi_{0}$ and $\alpha_{0}$ values.

**Algorithm A1** Algorithm of Lua script checking that both GNSS receivers are in RTK fix state
1: **while** True **do** 2: **if** *gps:num_sensors()* = 2 **then** 3: **if** *gps:status(0)* = GPS_OK_FIX_3D_RTK_FIXED *gps:status(1)* = GPS_OK_FIX_3D_RTK_FIXED **then** 4: Set *Script1* servo output to 2000 5: **else** 6: Set *Script1* servo output to 1000 7: **end if** 8: **else** 9: Set *Script1* servo output to 1000 10: **end if** 11: Wait 10 ms 12: **end while**

The modifications to the motion controller operation in different states are presented in Algorithm A2. These modifications affected two states: STR_RUN and STR_W_FIX. In the STR_RUN state, previously, the motion controller stored the camera pan and tilt setpoints evaluated by ArduPilot firmware and switched to STR_TARGET.

Currently, it first analyzes ArduPilot servo output related to relative targeting mode. If the targeting is performed using the standard ROI method (generally while capturing the first part of the equipment after arriving at a new keypoint of the inspection route), the state machine switches to the STR_W_FIX state. Otherwise, the camera pan and tilt setpoints according to (1) and (2) switch directly to STR_TARGET state because, in relative targeting mode, there is no need to know the robot’s position and heading after to target the desired equipment after the first angle was captured using the ROI method.

In the STR_W_FIX, the motion controller cyclically reads out camera orientation setpoints and waits until both GNSS receivers reach the RTK Fix state. Commonly, this state is immediately passed because, in case of robust communication between the RTK base, the GNSS receivers of the robot spend more than 99% of the inspection mission time in RTK Fix mode (as observed during experimental studies described in Section 4.1). At the same time, if the desired GNSS quality was not reached before the predefined time limit (in current research, it was configured to 10 s), the motion controller raises a special Skip flag and switches back to STR_RUN. This flag prevents the robot from targeting using the relative method if $\varphi_{0}$ and $\alpha_{0}$ were not correctly evaluated. Such behavior is motivated by the assumption that entirely skipping some parts of equipment is better than collecting data with an incorrectly targeted camera, as such data should be excluded from further processing anyway.

The modification to the state machine described above provides all the functionality necessary for implementing the relative targeting method. At the same time, it also requires support from mission generation software. Algorithm A3 demonstrates how relative targeting is represented in ArduPilot’s mission, starting from arrival to a keypoint.

**Algorithm A2** Modifications made in motion control algorithm to implement relative targeting method
1: **switch** (Motion controller’s state machine) 2: **case** STR_INIT**:** 3: … 4: **case** STR_LIFT**:** 5: … 6: **case** STR_RUN**:** 7: … 8: **if** Camera trigger servo level is valid and above 1500 **then** 9: **if** Relative targeting mode servo level is valid and below 1500 **then** 10: Switch ArduRover to Hold mode 11: Lower Skip flag 12: Start timeout timer for STR_W_FIX state 13: Switch the state machine to STR_W_FIX state 14: **else** 15: **if** Skip flag is lowered **then** 16: Set pan and tilt platform setpoints to $\varphi_{r_{n}}=\varphi_{0}+\varphi_{n}$ and $\alpha_{r_{n}}=\alpha_{0}+\alpha_{n}$, where $\varphi_{n}$ and $\alpha_{n}$ are decoded from dedicated S.Bus servo channels 17: Start timeout timer for STR_TARGET state 18: Switch the state machine to STR_TARGET state 19: **end if** 20: **end if** 21: **end if** 22: **case** STR_W_FIX**:** 23: **if** Pan/Tilt servo outputs are valid **then** 24: Set pan and tilt platform setpoints to the values evaluated using the corresponding servo outputs. 25: **end if** 26: **if** *Script1* servo output is valid and above 1500 **then** 27: Store pan and tilt platform setpoints into $\varphi_{0}$ and $\alpha_{0}$ 28: Start timeout timer for STR_TARGET state 29: Switch the state machine to STR_TARGET state 30: **else** 31: **if** Time limit reached **then** 32: Raise Skip flag 33: Switch the state machine to STR_RUN state 34: **end if** 35: **end if** 36: **case** STR_TARGET**:** 37: … 38: **case** STR_CAPTURE**:** 39: … 40: **case** STR_DEBLOCK**:** 41: … 42: **end switch**

**Algorithm A3** An example of ArduPilot mission’s part implementing relative targeting
**Require:** $S_{r}$—the identifier of the servo representing relative targeting mode (1000—ROI targeting, 200—relative targeting). **Require:** $S_{id}$—the identifier of the servo representing the keypoint identifier. **Require:** $S_{sid}$—the identifier of the servo representing the part of equipment captured from the keypoint. **Require:** $S_{\varphi }$—the identifier of the servo representing the yaw setpoint relative to direction on $r_{0}$. **Require:** $S_{\alpha }$—the identifier of the servo representing the pitch setpoint relative to direction on $r_{0}$ **Require:** $N_{id}$—the keypoint identifier. **Require:** $r_{0_{lat}}$, $r_{0_{lon}}$, $r_{0_{ralt}}$—latitude, longitude, and relative altitude of the equipment part $r_{0}$. 1: DO_SET_SERVO $S_{r}$, 1000 2: DO_SET_SERVO $S_{id}$, $2\cdot N_{ID}$ 3: DO_SET_SERVO $S_{sid}$, 2·0 4: DELAY 1.5 s 5: DO_SET_ROI $r_{0_{lat}}$, $r_{0_{lon}}$, $r_{0_{ralt}}$ 6: DO_DIGICAM_CONTROL 7: DELAY 1 s 8: DO_SET_SERVO $S_{r}$, 2000 9: DO_SET_SERVO $S_{sid}$, 2·1 10: DO_SET_SERVO $S_{\varphi }$, $\frac{500\varphi_{1}}{\varphi_{max}}+1500$ 11: DO_SET_SERVO $S_{\alpha }$, $\frac{500\alpha_{1}}{\alpha_{max}}+1500$ 12: DO_DIGICAM_CONTROL 13: DELAY 1 s 14: DO_SET_SERVO $S_{r}$, 1000

The example starts from switching to the ROI targeting mode, required to determine $\varphi_{0}$ and $\alpha_{0}$. Then, mission commands 2–3 pass the keypoint and equipment part identifiers to the motion controller that will re-transmit them further to the camera control script (Figure A1). These identifiers are multiplied by two to avoid any rounding problem during conversion between ArduPilot’s and S.Bus servo signal ranges.

The fourth mission command implements the delay required to stabilize yaw after the robot receives the stop command (Figure 9). It should be noted that this delay depends on the robot’s inertia and control law of the motion control level. Thus, it should be determined for each type of MAD robot.

Mission commands 5–7 target the first part of the equipment that should be captured from the keypoint using the ROI method and starting data acquisition. It is recommended to use DO_SET_ROI with relative altitude to minimize pitch error caused by GNSS-based altitude estimation, which is generally less precise than for horizontal coordinates.

The eighth command switches the motion controller to the relative targeting mode, while command 9 changes the current equipment part identifier to the following. Commands 10–11 define relative setpoints for targeting on $r_{1}$ equipment part. These setpoints are pre-evaluated during the mission generation procedure.Two DO_SET_SERVO commands are used instead of a single DO_MOUNT_CONTROL because relative setpoints need up to two times more range than absolute ones. Therefore, they should be coded in ArduPilot’s servo values with a different scaling ratio than general pan/tilt servo outputs. Implementing relative camera movement with two DO_SET_SERVO commands is one of the reasons why the relative targeting method results in large missions, as noted at the end of Section 5.1.

Commands 12–13, similarly to the ROI method, initiate camera targeting and data acquisition, while the last command switches the motion controller to ROI targeting mode.
